# The clinical challenges of homologous recombination proficiency in ovarian cancer: from intrinsic resistance to new treatment opportunities

**DOI:** 10.20517/cdr.2023.08

**Published:** 2023-07-28

**Authors:** Teresa Zielli, Intidhar Labidi-Galy, Maria Del Grande, Cristiana Sessa, Ilaria Colombo

**Affiliations:** ^1^Service of Medical Oncology, Oncology Institute of Southern Switzerland (IOSI), EOC, Bellinzona 6500, Switzerland.; ^2^Department of Oncology, Geneva University Hospitals, Geneva 1205, Switzerland.; ^3^Department of Medicine, Center of Translational Research in Onco-Hematology, Geneva 1205, Switzerland.

**Keywords:** PARP inhibitors, homologous recombination proficiency, ovarian cancer, PARP inhibitor resistance, replication stress

## Abstract

Ovarian cancer is the most lethal gynecologic cancer. Optimal cytoreductive surgery followed by platinum-based chemotherapy with or without bevacizumab is the conventional therapeutic strategy. Since 2016, the pharmacological treatment of epithelial ovarian cancer has significantly changed following the introduction of the poly (ADP-ribose) polymerase inhibitors (PARPi). *BRCA1/2* mutations and homologous recombination deficiency (HRD) have been established as predictive biomarkers of the benefit from platinum-based chemotherapy and PARPi. While in the absence of HRD (the so-called homologous recombination proficiency, HRp), patients derive minimal benefit from PARPi, the use of the antiangiogenic agent bevacizumab in first line did not result in different efficacy according to the presence of homologous recombination repair (HRR) genes mutations. No clinical trials have currently compared PARPi and bevacizumab as maintenance therapy in the HRp population. Different strategies are under investigation to overcome primary and acquired resistance to PARPi and to increase the sensitivity of HRp tumors to these agents. These tumors are characterized by frequent amplifications of Cyclin E and MYC, resulting in high replication stress. Different agents targeting DNA replication stress, such as ATR, WEE1 and CHK1 inhibitors, are currently being explored in preclinical models and clinical trials and have shown promising preliminary signs of activity. In this review, we will summarize the available evidence on the activity of PARPi in HRp tumors and the ongoing research to develop new treatment options in this hard-to-treat population.

## INTRODUCTION

Ovarian cancer (OC) is the deadliest gynecological tumor and the 13th leading cause of cancer mortality in the United States. In 2021, 21,410 new cases of OC were estimated, with 13,770 deaths due to this disease^[1]^. Among malignant ovarian tumors, epithelial ovarian cancer (EOC) constitutes the majority, with high-grade serous ovarian cancer (HGSOC) being the most common histological subtype^[2]^. The diagnosis of HGSOC commonly occurs at an advanced stage due to the consequence of non-specific symptoms and a lack of effective screening strategies^[3]^. The standard treatment combines optimal debulking surgery and platinum-based chemotherapy. Nevertheless, the majority of patients develop recurrence and the efficacy of subsequent lines of treatment decreases over time^[4]^. Maintenance treatment was introduced to delay disease progression after first-line treatment. The antiangiogenic agent bevacizumab has been the first targeted agent to receive authority approval in first-line treatment of International Federation of Gynecology and Obstetrics (FIGO) stage III and IV EOC^[5,6]^. Subsequently, the development of poly (ADP-ribose) polymerase inhibitors (PARPi) has represented a paradigm change in the treatment of EOC and they are now incorporated into the standard of care treatment^[7]^.

## PARPi IN OVARIAN CANCER

Poly (ADP-ribose) polymerases (PARPs) are responsible for the detection of single-strand DNA breaks (SSBs) and the recruitment of DNA repair factors at the site of DNA damage^[8]^. Following PARP inhibition, SSBs are not properly repaired and PARP is trapped and the replication fork stalled with the subsequent occurrence of DNA double-strand breaks (DSBs)^[8,9]^. These DNA damages activate, leading to an error-free DNA damage repair.

Approximately 15% of patients with EOC harbor a germline mutation in *BRCA1/2* (*gBRCA*)^[10]^. *BRCA1* and *BRCA2* are tumor suppressor genes involved in multiple cellular pathways, such as transcription, cell cycle regulation, and maintenance of genome integrity^[11]^. Specifically, they are involved in DNA DSB repair and are essential for the HRR pathway.

PARPi are particularly active in cancer cells with alterations in HRR, such as *BRCA1* or *BRCA2* pathogenic mutations*,* through the well-known mechanism of “synthetic lethality”^[12,13]^. With the loss of HRR function, cells repair DSBs via the non-homologous end joining (NHEJ), resulting in genomic instability and cell death.

PARPi have been studied in high-grade EOC in different phase II and III clinical trials as maintenance and treatment strategies, leading to approvals by health authorities for different indications^[14-23]^.

## HOMOLOGOUS RECOMBINATION DEFICIENCY

The most disruptive form of DNA damage is the DNA DSBs, which are repaired by NHEJ and homologous recombination^[24]^. When DNA damage occurs, different enzymes are activated, including the MRE11-RAD50-NBS1 complex (MRN), the DNA damage kinase ataxia-telangiectasia mutated (ATM), and the ataxia telangiectasia and Rad3-related (ATR). Subsequently, DNA repair is activated by *CHEK2*, *BRCA1*, *BRCA2*, and *RAD51*. Other important effectors of the HRR pathway are *PALB2* and *BRIP1*^[11]^. A defect in the HRR process determines a condition called homologous recombination deficiency (HRD) that occurs as a consequence of germline or somatic mutations, or epigenetic modifications of different genes involved in this pathway.

Almost 50% of high-grade EOC are characterized by HRD, with 12%-15% and 5%-7% harboring germline or somatic mutations in *BRCA1/2*, respectively^[25-27]^. However, HRD also occurs due to germline or somatic mutations or epigenetic events in other HRR genes, and not all mechanisms underscoring HRD have been characterized yet^[27,28]^.

### HRD assays and their limitations

Different types of assays, referred to as “HRD tests”, are available to select patients more likely to benefit from PARPi^[28]^. HRD can be measured using different strategies, which can be categorized into four main groups and each of them has its own limitations^[28,29]^.

#### Germline or somatic mutations in HRR genes

Next-generation sequencing (NGS) of DNA extracted from peripheral blood or saliva is used to detect germline pathogenic variants of genes involved in HRR. In patients without a g*BRCA* mutation, a somatic test on formalin-fixed paraffin-embedded (FFPE) tissue is recommended, given ~5%-7% of EOC harbor a somatic mutation in *BRCA1/2*^[27,30,31]^. In addition to *BRCA1/2* mutations, other causes of HRD include promoter methylation of *BRCA1* (10%) or *RAD51C* (3%) or mutations in other genes such as ATM or ATR (~2%), CDK12 (~3%), *BRIP1*, *RAD51C*, *RAD51D*, *PALB2*, and BARD1 (~5%)^[30]^. Different studies demonstrated similar efficacy of PARPi in patients with somatic compared to germline *BRCA* mutations^[20,32,33]^. Mutations in HRR genes other than *BRCA* are rare, with only limited data available on the benefit of PARPi in this setting. A retrospective analysis of the PAOLA-1 trial demonstrated that the presence of non-*BRCA* HRR gene mutations, contrary to HRD positive status, was not predictive of progression-free survival (PFS) benefit^[34]^. The exploratory analysis of the ARIEL2 trial showed that the presence of loss of heterozygosity (LOH) in *BRCA* wild-type tumors was a more sensitive biomarker of response compared to other HRR gene mutations or *BRCA1* and *RAD51C* methylation^[23,33]^. An important challenge in the identification of germline and somatic mutations is the definition of the functional and clinical meaning of variants of uncertain significance (VUS) and their correlation with benefit from PARPi^[35]^. Despite the work of the ENIGMA (Evidence-based Network for the Interpretation of Germline Mutant Alleles) consortium to reclassify many VUS as benign or malignant, the absolute number of individuals receiving an inconclusive *BRCA1/2* test result is still significant^[36]^.

#### Genomic “scars” and mutational signatures

HRD causes genomic instability with a progressive accumulation of genomic aberrations that result in “scars” that can be measured with specific assays. These assays do not look for the cause of HRD, but to the consequences of having a defective HRR pathway. Several investigations have been conducted using single nucleotide polymorphism arrays to define a signature of genomic instability associated with *BRCA* mutations. The three major types of genomic “scars” associated with HRD are LOH, telomeric allelic Imbalance (TAI) and large-scale state transitions (LST)^[37-39]^. Two commercially available genomic instability assays have been developed: the myChoice® CDx from Myriad Genetics and the FoundationFocus^TM^ CDx_BRCA LOH_ by Foundation Medicine. The myChoice® CDx assay evaluates tumor *BRCA1/2* mutation (t*BRCA*), LOH, TAI and LST with a genomic instability score (GIS) cut off at 42^[15,16]^. The FoundationFocus^TM^ assay evaluates t*BRCA* and genomic LOH, with an initial cut-off for LOH positivity established at 14%^[26]^. This cut-off was initially prospectively evaluated in the ARIEL2 trial^[23]^, but subsequently changed to 16% according to the ARIEL3 trial results, which corresponds to the cut-off currently used in the available assay^[22]^.

One of the major limitations of these genomic instability assays is the inability to define the current HRD status. Genomic scars can persist even if tumor cells have restored HRR through mechanisms, such as the occurrence of reversion mutations in *BRCA* and other HRR-related genes^[40-42]^. Notably, different cut-offs have been employed to define the presence of HRD in the different clinical trials evaluating the role of PARPi in EOC, and direct comparisons among the two approved assays are not available. Other limitations of the currently commercially available HRD tests are the limited reproducibility, their availability and cost. Several research groups are validating academic tests with the aim of overcoming such limitations and preliminary results have been recently presented^[43,44]^.

Mutational signatures can also be used to identify the presence of HRD. This method is based on the assumption that several mutational processes, such as smoking, carcinogens, ultraviolet radiation or defects in DNA repair mechanisms, cause somatic mutations and each of them generates a specific mutational signature^[45]^. Signature 3 is the one associated with *BRCA* mutation and *BRCA1* promoter methylation in different solid tumors, including ovarian cancer^[45-47]^. Hillman *et al.* conducted a retrospective whole genome sequencing (WGS) of HGSOC samples and demonstrated that the presence of signature 3 correlates with better prognosis and response to platinum agents^[48]^. However, the most common genomic testing platform used in clinical practice are targeted sequencing panels with a number of identifiable mutations too small for the identification of a specific signature. To overcome this limitation, Gulhan *et al.* have developed a bioinformatics algorithm (Signature Multivariate Analysis-SigMA), which can identify the mutational signature correlated with HRD using genetic panels^[49]^. In their analyses, cancer cell lines with signature 3 showed greater sensitivity to PARPi, irrespective of the *BRCA* mutational status, not only in breast and ovarian cancers but also in other tumor types^[49]^.

Similar to other genomic “scars” assays, the identification of a mutational signature linked to defects in DNA repair pathways also remains a historical representation of HRD and does not reflect the actual presence of the mechanism of resistance to PARPi. Another major limitation is the need for fresh frozen material to perform these assays. Despite being a promising approach, it lacks clinical validation^[28]^.

#### Functional assays

Functional assays are a dynamic assessment of the HRR status^[28]^. In the presence of DNA DSBs, RAD51 forms molecular complexes (foci) at the DNA damage site, which are visible by immunofluorescence microscopy. It has been shown that cell lines with HRD are not able to form RAD51 foci when DNA damage occurs and this represents a functional reflection of a defect in the homologous recombination pathway^[50-52]^. Blanc-Durant *et al.* evaluated a RAD51 functional assay in tumor samples from patients enrolled in the randomized neoadjuvant CHIVA trial assessing platinum-based chemotherapy +/- nintedanib^[53,54]^. They demonstrated that RAD51-deficient ovarian tumors had better overall response rates (ORR) to neoadjuvant chemotherapy and median PFS. Notably, among *BRCA1/2* mutated EOC, patients with RAD51-proficient tumors had a poorer response to chemotherapy^[53,54]^. RAD51 measurement as a surrogate for HRD has several limitations. Not all mechanisms underlying PARPi sensitivity involve a deficiency in RAD51. When PARPi sensitivity is driven by ATM or MRN complex alterations, RAD51 assay is not able to detect it because these mechanisms preserve the formation of RAD51 foci^[50]^. Similarly, if the resistance to PARPi is due to a RAD51-independent mechanism, such as mutations in PARP1, it is not identified by a RAD51 assay^[55]^. Additionally, the assay relies on counting and quantifying many foci, which may result in substantial inter-observer variability^[56]^. The available data on RAD51 functional assays are retrospective and performed on a limited number of samples^[56]^. A prospective trial that stratifies patients based on the presence of RAD51 foci is needed to validate its clinical utility.

## PARPi IN HRp TUMORS

EOC associated with *gBRCA* mutations has different clinical features, such as earlier age of onset, longer survival, higher rate of visceral metastasis (e.g., liver, spleen), higher response rates to platinum and non-platinum chemotherapy (e.g., pegylated liposomal doxorubicin), and increased sensitivity to PARPi^[57-60]^. It has become evident that many HGSOCs share the same biological and clinical features even when a germline or somatic *BRCA* mutation is not detected, a condition called “BRCAness”^[27]^. In contrast, patients with HRp tumors have worse PFS and OS compared to patients with HRD tumors^[61,62]^. A retrospective analysis of 1,271 patients with EOC showed that patients with HRp tumors have an older median age at diagnosis, have more commonly non-serous histology, require a higher number of neoadjuvant chemotherapy cycles to be considered for interval debulking surgery, and are less sensitive to platinum-based chemotherapy^[62,63]^.

Homologous recombination-proficient EOC also harbors different genomic features. Amplification of cyclin E1 (CCNE1) is one of the best characterized genomic alterations linked to resistance to platinum-based chemotherapy and is mutually exclusive with *BRCA1/2* dysfunction^[64,65]^. CCNE1 encodes the cell-cycle regulator cyclin E1, which is required for the cell-cycle progression from G1 to S through the p21-p27-cyclin E-CDK2 pathway. CCNE1 amplification raises cyclin E levels, leading to abrogation of the G1-S cell cycle checkpoint and reduction of the G1 phase^[66]^. This results in high replicative stress (RS) due to aberrant firing of the replication origin^[66]^. MYC amplification is also found in HRp cancer and correlates with increased RS. MYC-amplified cancer cells are characterized by inadequate reduction/oxidation balance with accumulation of reactive oxygen species, accelerated entry in S phase and impairment in the expression of genes implicated in purine and pyrimidine biosynthesis and in the regulation of the HR pathway^[66,67]^.

While recent clinical trials have clearly proved the activity of PARPi in the presence of *BRCA* mutation and/or HRD, their role in the HRp population is less understood.

In the first-line setting, three trials have investigated the role of maintenance treatment with a PARPi following a complete or partial response (CR/PR) to chemotherapy [Table 1]. The PRIMA trial is a phase 3 randomized trial of niraparib *vs.* placebo as maintenance treatment in patients with high-risk stage III (residual disease after debulking surgery, inoperable stage III and patients who have received neoadjuvant chemotherapy) and stage IV high-grade EOC. Although an improvement in median PFS in the overall population regardless of HRD status [defined as *BRCA* mutation or/and a GIS score ≥ 42 (myChoice® assay)] was obtained with niraparib, in the HRp population, the benefit was smaller^[2]^. The phase III PAOLA-1 trial investigated the addition of olaparib to bevacizumab as maintenance treatment in newly diagnosed, stage III-IV high-grade EOC after response to first-line chemotherapy^[15]^. This study demonstrated a statistically and clinically significant improvement in PFS and overall survival (OS) in the overall population, in the HRD-positive/t*BRCA* and HRD-positive/*BRCA*wt groups, but with no difference in the HRp population^[15,68]^. Recently, the phase 3 ATHENA-MONO trial has confirmed the activity of rucaparib regardless of the HRD status, measured as LOH using the FoundationOne CDx NGS assay^[69]^.

**Table 1:** Results of the main randomized phase 2 and 3 clinical trials investigating PARPi in EOC, including HRp population

**Trial**	**PRIMA**	**PAOLA-1**	**ATHENA-MONO**	**VELIA**	**NOVA**	**ARIEL3**	**AVANOVA**
Treatment	Niraparib *vs.* placebo	Olaparib + bev *vs.* placebo + bev	Rucaparib *vs.* placebo	Veliparib + cht → veliparib *vs.* cht + placebo → placebo^&^	Niraparib *vs.* placebo	Rucaparib *vs.* placebo	Niraparib + bev *vs.* niraparib + placebo
Setting	First line maintenance	First line maintenance	First line maintenance	First line with chemo and maintenance	Platinum-sensitive recurrence maintenance	Platinum-sensitive recurrence maintenance	Platinum sensitive recurrence, ≤ 1 prior non-platinum lines
PFS ITT (months)	13.8 *vs.* 8.2 (HR 0.62)	22.1 *vs.* 16.6 (HR 0.59)	20.2 *vs.* 9.2 (HR 0.52)	23.5 *vs.* 17.3 (HR 0.68)	NA	10.8 *vs.* 5.4 (HR 0.36)	11.9 *vs.* 5.5 (HR 0.35)
PFS *BRCA*mut (months)	22.1 *vs.* 10.9 (HR 0.40)	37.2 *vs.* 21.7 (HR 0.31)	NR *vs.* 14.7 (0.40)	34.7 *vs.* 22 (HR 0.44)	21 *vs.* 5.5 (HR 0.27)	16.6 *vs.* 5.4 (HR 0.23)	14.4 *vs.* 9 (HR: 0.49)
PFS HRDpos (months)	21.9 *vs.* 10.4 (HR 0.43)	37.2 *vs.* 17.7 (HR 0.33)	28.7 *vs.* 11.3 (HR 0.47)	31.9 *vs.* 20.5 (HR 0.57)	12.9 *vs.* 3.8 (HR 0.38)	13.6 *vs.* 5.4 (HR 0.32)	11.9 *vs.* 6.1 (HR 0.38)
PFS HRDpos/*BRCA*wt (months)	19.6 *vs.* 8.2 (HR 0.50)	28.1 *vs.* 16.6 (HR 0.43)	20.3 *vs.* 9.2 (HR 0.58)	NA	9.3 *vs.* 3.7 (HR 0.38)	^*^9.7 *vs.* 5.4 (HR 0.44)	11.9 *vs.* 4.1 (HR 0.19)
PFS HRp (months)	8.1 *vs.* 5.4 (HR 0.68)	16.9 *vs.* 16 (HR 0.92)	12.1 *vs.* 9.1 (HR 0.65)	15 *vs.* 11.5 (HR 0.81)	6.9 *vs.* 3.8 (HR 0.58)	^**^6.7 *vs.* 5.4 (HR 0.58)	11.3 *vs.* 4.2 (HR 0.40)
HRD test	Myriad MyChoice®	Myriad myChoice HRD Plus®	FoundationOne CDx	Myriad myChoice HRD CDx®^§^	Myriad MyChoice HRD®	Foundation Medicine T5 NGS	Myriad MyChoice HRD®
Role of HRD score	Stratification factor	Prespecified subgroup analysis	Stratification factor	Prespecified subgroup analysis	Prespecified subgroup analysis	Stratification factor	Stratification factor

* BRCAwt, high LOH; ^**^BRCAwt, low LOH; ^&^Data of the veliparib combination only arm are not reported; ^§^Initially the cut off for HRD positivity was ≥ 33, subsequently revised to ≥ 42. bev: Bevacizumab; BRCAmut: BRCA mutated; BRCAwt: BRCA wild type; cht: chemotherapy; EOC: epithelial ovarian cancer; HR: hazard ratio; HRD: homologous recombination deficiency; HRp: homologous recombination proficient; ITT: intention-to-treat; LOH: loss of heterozygosity; NA: not available; NR: not reach; PARPi: poly (ADP-ribose) polymerase inhibitors; PFS: progression-free survival; *vs.*: versus.

The combination of veliparib with chemotherapy containing platinum compound in stage III/IV EOC was evaluated in the phase 3 VELIA trial^[70]^. Patients were randomized in three different arms: chemotherapy plus placebo followed by placebo maintenance, chemotherapy plus veliparib followed by placebo maintenance, or chemotherapy plus veliparib followed by veliparib maintenance. Veliparib, administered concomitantly with chemotherapy and as maintenance treatment, improved patients’ outcomes (PFS from the start of first-line chemotherapy) in the three prespecified subgroups: *BRCA* mutated, HRD positive and the overall population. The HRD population includes patients with *BRCA* mutated tumors and/or a GIS score ≥ 42 (myChoice® assay). In the HRp subgroup, the effectiveness of the standard of care treatment was not enhanced by the addition of velaparib^[70]^.

In the platinum-sensitive recurrent setting, niraparib and rucaparib have shown effectiveness in the different biomarker subgroups. However, as described for the first line setting, there is a different magnitude of benefit, with higher efficacy observed in the *BRCA* mutated population followed by the HRD non-*BRCA* mutated and the HRp subgroups. This was clearly observed in the phase 3 NOVA trial, where the role of niraparib versus placebo as maintenance therapy in platinum-sensitive recurrent high-grade EOC was assessed^[20]^. HRD positivity was defined when the GIS by myChoice® score was ≥ 42 or a *BRCA1/2* mutation was present. To be noted, a potential detrimental effect on OS has been reported, and final data are awaited^[20,71]^.

In the same setting, the ARIEL3 trial evaluated rucaparib as a maintenance treatment, showing an improvement in median PFS in the intention-to-treat population in HRD population, defined as high-LOH (LOH score ≥ 16%) or *BRCA* mutated. As expected, the PFS benefit was greater in patients with LOH high-*BRCAwt* compared to LOH low patients^[22]^.

PARPi have also been investigated as a line of treatment in the ARIEL2^[23]^ and QUADRA trials^[21]^. QUADRA was a single-arm phase II study where niraparib was administered as monotherapy in patients with recurrent HGSOC after the failure of three or more previous regimens. HRD tumors included *BRCA* mutated and/or myChoice® HRD score ≥ 42. The ORR was 28%, with a median duration of response similar in the different subgroups of patients. Median OS was 26.0 months (95%CI, 18.1-not estimable), 19.0 months (14.5-24.6), and 15.5 months (11.6-19.0) in the *BRCA* mutated, HRD-positive, and HRp populations^[21]^. The phase 2 ARIEL2 trial assessed the role of rucaparib as treatment. Part 1 of the trial included patients with platinum-sensitive high-grade EOC treated with one or more prior chemotherapy regimens. The analysis has been performed according to three prespecified subgroups: *BRCA* mutant, LOH high and *BRCAwt* (cut off for LOH high 14%), or LOH low. Median PFS with rucaparib treatment was 12.8 (95%CI 9.0 to 14.7), 5.7 months (5.3 to 7.6) and 5.2 months (3.6 to 5.5) in the *BRCA* mutant, LOH high and LOH low patients, respectively. Part 2 of the trial enrolled patients with any platinum-free interval disease who had completed 3 to 4 prior chemotherapies. Patients with *BRCA* mutated tumors had a median PFS of 7.8 months and an ORR of 45.7% (95%CI, 37.2 to 54.3). In contrast, in the LOH-high and LOH-low groups, median PFS was 4.3 and 4.0 months and ORR was 16.7% (95%CI, 11.2 to 23.5) and 7.7% (95%CI, 4.2 to 12.9), respectively^[23]^.

The AVANOVA2 is a phase 2 trial that compared niraparib versus the combination of niraparib plus bevacizumab in patients with platinum-sensitive recurrent ovarian cancer. In the prespecified subgroup analysis, a longer PFS was observed with the combination regardless of the HRD status^[72]^.

**Figure 1:**
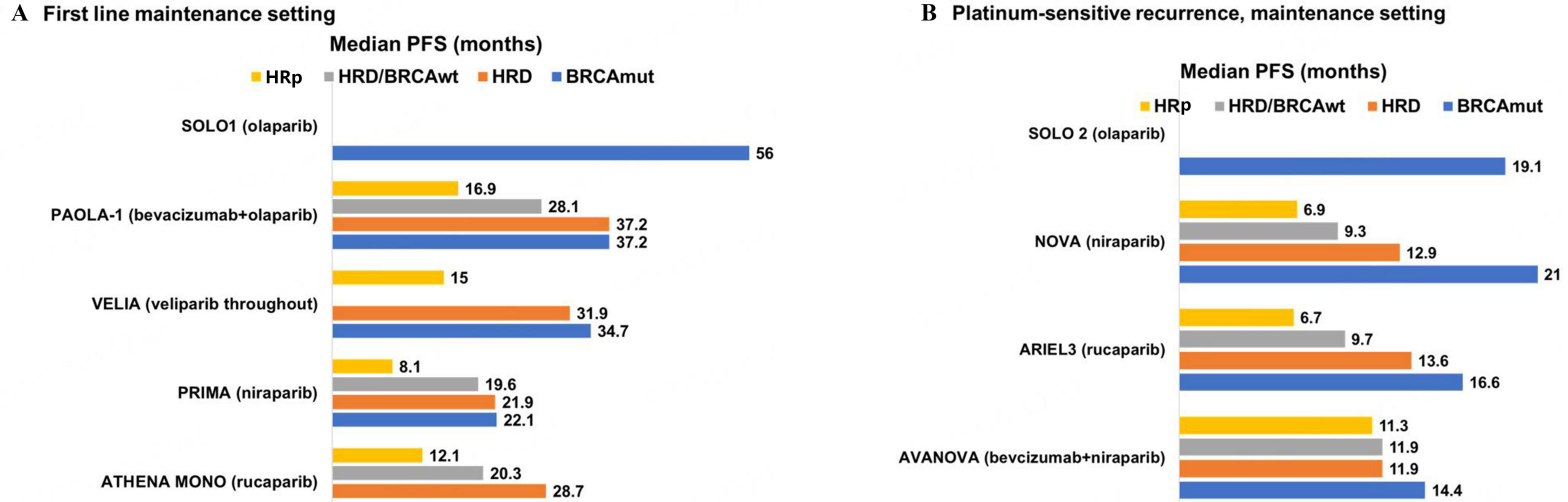
Indirect comparison of PFS with PARPi maintenance according to *BRCA* or HRD/LOH status in the main randomized phase 3 trials. In VELIA trial, PFS was calculated from the start of chemotherapy. ATHENA MONO: PFS in BRCA mutated population is not reached. BRCAmut: BRCA mutated; BRCAwt: BRCA wild type; HRD: homologous recombination deficiency; HRp: homologous recombination proficiency; LOH: loss of heterozygosity; PARPi: poly (ADP-ribose) polymerase inhibitors; PFS: progression-free survival; VELIA: PSF in HRD/BRCAwt not reported.

Results of the main phase 2 and 3 trials of PARPi in EOC that have included HRp patients are summarized in Table 1 and Figure 1A and B.

## MAINTENANCE TREATMENT IN HRp TUMORS: PARPi OR ANTIANGIOGENIC AGENTS

The antiangiogenic agent bevacizumab has demonstrated an improvement in PFS when used with chemotherapy and as maintenance in first-line settings, but also in platinum-sensitive recurrent disease^[5,6,73,74]^. The GOG-218 is a 3-arm phase 3 trial assessing the efficacy of bevacizumab added to first-line chemotherapy in patients with stage III and residual disease after surgery and stage IV EOC. Patients were randomized to receive six cycles of carboplatin, paclitaxel and placebo followed by placebo (arm 1), or the same chemotherapy with the addition of bevacizumab (15 mg/kg) followed by placebo maintenance (arm 2) or carboplatin and paclitaxel with bevacizumab and bevacizumab maintenance up to 22 total administrations (arm 3). Treatment with bevacizumab concurrent plus maintenance reduced the risk of disease progression by 28% (median PFS 14.1 *vs.* 10.3 months; HR = 0.717; 95%CI, 0.625 to 0.824)^[5]^. The difference in OS was noted only in the subgroup of patients with stage IV disease. A retrospective analysis evaluated the impact of HRR gene mutations (such as *ATM, ATR, BARD1, BRCA1, BRCA2, BRIP1, CHEK2, PALB2, RAD51C, RAD51D, and others*) on OS, platinum and bevacizumab sensitivity (comparing arm1 and 3)^[75]^. This analysis demonstrated that mutations in HRR might be correlated with a better PFS and OS in patients with EOC, but bevacizumab did not result in a different benefit according to the mutational status^[75]^.

A small retrospective study analyzed the role of CCNE overexpression in predicting the benefit of bevacizumab in 57 patients with platinum-sensitive recurrence of OC. 45.6% of the patients presented CCNE1 overexpression and 15 (62.5%) were treated with chemotherapy and bevacizumab and 11 (33.3%) received chemotherapy alone. Among patients with CCNE1 overexpression, the ORR was 100% and 50% in the group treated with or without bevacizumab, respectively. The PFS was higher in patients with CCNE1 overexpression who received bevacizumab (16.3 *vs.* 7.1 months, *P* = 0.010)^[76]^.

To date, no randomized trials comparing PARPi plus bevacizumab *vs.* PARPi alone, or PARPi *vs.* bevacizumab are available. Hettle *et al.* performed a population-adjusted indirect treatment comparison among the high-risk population of the PAOLA-1 trial matched to the PRIMA cohort^[77]^. The authors compared the relative efficacy of olaparib plus bevacizumab (group 1), bevacizumab alone (group 2), niraparib (group 3) and placebo (group 4). Median PFS in the biomarker unselected population was 21.4 months (95%CI, 19.2 to 22.1) in group 1, 16.0 months (95%CI, 14.3 to 17.7) for patients in group 2, 13.8 months (95%CI, 11.5 to 15.3) for patients in group 3 and 8.1 months (95%CI, 7.31 to 8.51) for patients in the placebo arm. In the HRD population, the greater advantage was achieved with the association of PARPi and bevacizumab than from PARPi alone or bevacizumab alone, with a median PFS of 36.0 months [95%CI, 23.2-not available (NA)] for patients in the olaparib plus bevacizumab arm, 17.6 months (95%CI, 14.7 to 19.6) for patients in the placebo plus bevacizumab arm, 22.0 months (95%CI, 19.3 to NA) for patients in the niraparib arm and 10.5 months (95%CI, 8.05 to 12.1) for patients in the placebo arm. Unfortunately, because of the lack of baseline data for the HRp subgroup of the PRIMA trial, it was not possible to repeat the same comparative analysis in the HRp population^[77]^.

The MITO-MANGO16 trial explored the effect of adding bevacizumab to chemotherapy in patients previously treated with the same antiangiogenic agent and experiencing the first platinum-sensitive recurrence. Bevacizumab beyond progression resulted in a better PFS 11.8 *vs.* 8.8 months (HR 0.51; 95%CI, 0.41 to 0.65). In a non-preplanned subgroup analysis, PFS was not improved in *BRCA* mutated patients^[78]^.

In the GOG-218 and MITO-MANGO16 trials, HRD tests have not been performed, and thus, an indirect comparison with the HRp populations is not possible. Notably, it is important to highlight that in the PARPi trials, the randomization occurred after the end of the chemotherapy and patients without at least a partial response were excluded, while in the bevacizumab trials, the randomization occurred at the time of initiation of first-line treatment. Thus, the comparison of the magnitude of PFS needs to account for this difference^[5,78]^.

The PAOLA-1 trial failed to show a benefit of adding olaparib to bevacizumab in the HRp subgroup (HR 0.92; 95%CI, 0.72 to 1.17)^[15]^. In the AVANOVA trial, the combination of niraparib and bevacizumab showed a statistically significant improvement in PFS, even in the HRp population (HR 0.40; 95%CI, 0.19 to 0.85)^[72]^. The main difference between these two studies is the control arm, that is bevacizumab in the PAOLA-1 trial and niraparib in the AVANOVA. Although a direct comparison among these trials is not feasible, these results raise the question if the HRp population could gain a higher benefit from the treatment with bevacizumab than a PARPi.

The ongoing MITO 25 trial (NCT03462212) might help in answering this question. This is a phase 1/2 trial evaluating the efficacy of carboplatin-paclitaxel and rucaparib maintenance versus carboplatin-paclitaxel-bevacizumab and bevacizumab plus rucaparib maintenance in HRD-positive HGSOC and of carboplatin-paclitaxel-bevacizumab and bevacizumab maintenance versus carboplatin-paclitaxel and rucaparib maintenance in HRp patients.

## INCREASING THE EFFICACY OF PARPi IN HRp TUMORS

**Figure 2:**
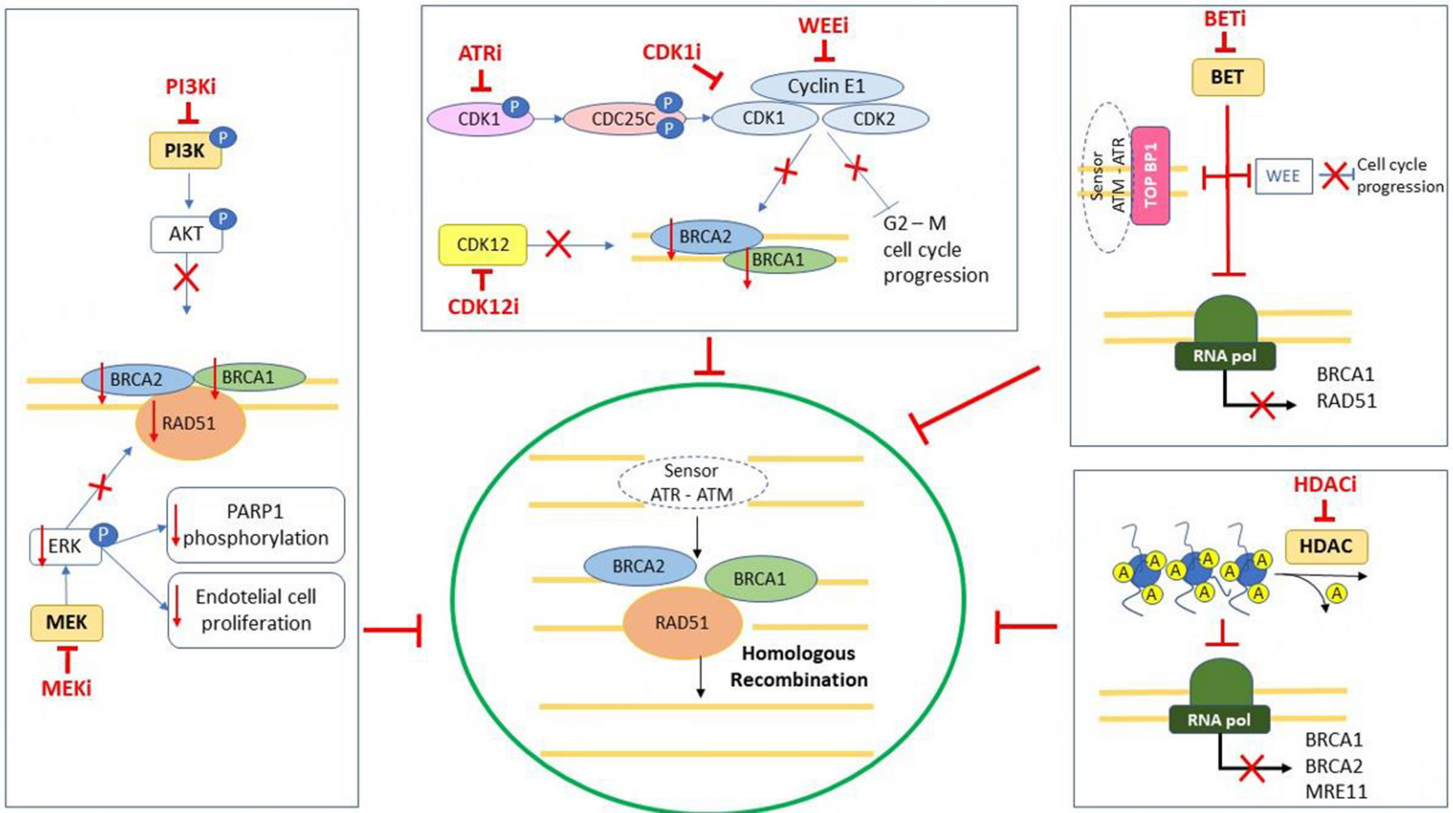
Cellular pathways that can be targeted to overcome PARPi resistance in HRp tumors. ATM: Ataxia-telangiectasia mutated; ATR: ataxia telangiectasia and Rad3-related; CDK1: cyclin-dependent kinase 1; CDK2: cyclin-dependent kinase 2; CDK12: cyclin-dependent kinase 12; ERK: extracellular signal-regulated kinase; HDAC: histone deacetylases; MEK: MAPK/ERK kinase; PARP: poly (ADP-ribose) polymerases inhibitors; PI3K: phosphatidylinositol-3 kinase; RNApol: RNA polymerase; TOPBP1: DNA topoisomerase II binding protein 1.

As previously described, the efficacy of PARPi is significantly different among HRD and HRp tumors. Thus, there is an unmet need to identify new treatment strategies to increase the activity of DNA-damaging agents in this patient population. Preclinical studies have shown that different pathways are involved in the regulation of the DDR^[79]^ [Figure 2]. The phosphoinositide 3-kinase (PI3K) and MEK pathways are involved in recognition of DNA damage and the promotion of homologous recombination, while the cyclin-dependent kinases (CDK) are involved in the control of the cell cycle progression and act in coordination with the DDR pathways^[80-83]^. Transcriptional and epigenetic regulation has a critical role in the downregulation of HRR genes, particularly due to the close link between the DDR pathway and chromatin remodeling by histone modifications^[79]^. Targeting these pathways has the potential to pharmacologically induce an HRD phenotype and might represent a powerful strategy to sensitize HRp EOC to PARPi. Several compounds have been evaluated as potential inducers of HRD and preliminary preclinical and clinical results are available [Table 2].

**Table 2:** Ongoing clinical trials of PARPi in combination with new agents in EOC

**Class of drugs**	**Phase**	**Combination**	**Population**	**N**
HDACi	I/II	Entinostat + olaparib	Platinum-refractory or resistant, HRp EOC	NCT03924245
	I	Belinostat + talazoparib	mBC, mCRPC, EOC progressed to at least one line of chemotherapy	NCT04703920
BETi	II	ZEN003694 + talazoparib	EOC PARPi resistant and platinum-sensitive (PFI > 6 months)	NCT05071937
PI3Ki	I	BKM120 + Olaparib or BYL719 and olaparib	TNBC and EOC, progressed after at least one prior platinum-based chemotherapy	NCT01623349
	I/II	CYH33 + olaparib	Solid tumors with any DDR gene or PIK3CA mutation, including PARPi and platinum-resistant EOC	NCT04586335
	Ib	Copanlisib + niraparib	Platinum-resistant EOC and *BRCA* mutated PARPi resistant EOC	NCT03586661
MEKi	I/II	Selumetinib + olaparib	Solid tumors with RAS pathway alterations and PARPi resistance EOC	NCT03162627
ATRi	I/II	BAY1895344 + niraparib	Part A: DDR deficiency solid tumor. Part B: platinum-resistant/refractory or PARPi-resistant EOC	NCT04267939
	I	M4344 + niraparib	PARPi resistant EOC	NCT04149145
	II	AZD6738 + olaparib	Recurrent EOC	NCT03462342
WEE1i	II	Adavosertib +olaparib	PARPi resistant EOC	NCT03579316
CDK12i	I	Dinaciclib + veliparib	Solid tumors	NCT01434316

ATRi: ATR inhibitors; BETi: BET inhibitor; CDK: cyclin-dependent kinases; EOC: epithelial ovarian cancer; HDACi: histone deacetylases inhibitor; HRp: homologous recombination proficient; mBC: metastatic breast cancer; mCRPC: metastatic castration-resistant prostate cancer; PARPi: poly (ADP-ribose) polymerase inhibitors; PFI: platinum-free interval; PI3Ki: phosphatidylinositol-3 kinases inhibitor; TNBC: triple-negative breast cancer; WEE1i: WEE1 inhibitors.

### Transcriptional regulators

Histone deacetylases (HDACs) have a key role in gene transcription, DNA replication and repair. Preclinical research has demonstrated that HDAC inhibitors reduce DNA repair by inhibiting HR genes, which, in turn, creates an HRD-like phenotype. This, along with a perturbed replication fork progression, leads to DNA DSBs, irreversible DNA damage, and finally cell death^[84]^. Gupta *et al.* studied the effects of olaparib and the HDAC inhibitor entinostatin in HRp ovarian cancer xenograft models, demonstrating that the combination reduces the peritoneal spread and prolongs survival^[85]^. A phase I/II study is ongoing assessing the safety and efficacy of olaparib combined with entinostat in patients with HRp ovarian cancer (NCT03924245).

The bromodomain and extraterminal (BET) protein BRD4 supports gene transcription and is implicated in the expression of proteins that regulate the cell cycle and the DDR. *BRCA* wild-type ovarian cancer cells treated with the BET inhibitor (BETi) JQ1 exhibit a downregulation of WEE1 and the DNA factor TOPBP1^[86,87]^. Moreover, the BETi INCB054329 directly represses the transcription of *BRCA1* and *RAD51* in cancer cells^[88]^. The association of olaparib and JQ1 suppresses the growth of HRp EOC xenografts, while there was no significant effect of either BETi or olaparib if used as a single agent^[88]^. More information on the efficacy of these combinations will become available from the phase II trial of the BETi, ZEN003694, combined with the PARPi, talazoparib, in patients with recurrent ovarian cancer progressed to prior PARPi (NCT05071937).

### PI3K and MEK inhibitors

Preclinical studies have shown that treatment with a phosphatidylinositol-3 kinases inhibitor (PI3Ki) decreases *BRCA1* expression and induces an increase of γ-H2AX, a marker of DNA damage^[80]^. *BRCA1/2* downregulation seems to be due to ERK-dependent activation of the erythroblast transformation specific (ETS) transcription factor, which inhibits *BRCA1* or *BRCA2* transcription, thereby resulting in HRD and concomitantly increasing the sensitivity to PARPi^[80]^. A phase 1b trial investigated the synergy between the PI3Ki alpelisib and olaparib in patients with breast and ovarian cancer. This study included 30 women with EOC (mainly HGSOC) and 93% had platinum resistant/refractory disease. This trial showed an ORR of 33% in *BRCA*wt platinum-resistant patients, while the ORR of olaparib or other PARPi as monotherapy in the same setting ranges from 3% to 10%^[89]^. An ongoing phase 3 trial is investigating alpelisib and olaparib versus chemotherapy of physician’s choice in patients with platinum-resistant *BRCA* wild type EOC (NCT04729387).

MEK inhibition decreased both the MRE11 and *RAD51* foci at DSBs as well as the *BRCA1* nuclear localization, with a consequent accumulation of DNA damage^[81]^. Moreover, the increased expression of PARP1 and the decreased vascularity increases the hypoxia^[81]^. It was shown that hypoxia induces transcriptional repression of *BRCA1* expression, with a consequent HRD status sustaining a greater sensitivity to PARPi^[90]^. Olaparib in combination with selumetinib has been investigated in an early-phase trial in patients with KRAS/NRAS mutant and *BRCA*wt solid tumors, including gynecological cancers. Among 12 evaluable patients, ORR was 17%, and clinical benefit rate (CBR) 33%^[91]^. Further investigations are needed to further understand the clinical activity of this combination in recurrent EOC.

### Cell cycle check-point inhibitors

An effective suppression of HR and a consequent sensitization to PARPi can also be achieved through the inhibition of CDK. Inhibition of CDK12 causes a reduced expression of *BRCA1*, *BRCA2*, and *RAD51*^[92]^. Inhibition of CDK1 blocks the DNA repair mechanism sustained by the HR pathway and selectively sensitizes cells to PARPi^[84]^. Xia *et al.* demonstrated a synergistic effect of CDK1 and PARP inhibitors in breast cancer cells proficient for BRCA^[93]^. Prexasertib is a checkpoint kinase 1 inhibitor (CHK1i) that showed efficacy in a phase 2 trial that enrolled 28 women with *BRCAwt* high-grade EOC. The majority (80%) had platinum-resistant or refractory disease. 8/24 evaluable patients achieved a PR (33%), and the median duration of treatment was 11.4 months^[94]^. Notably, CCNE1 amplification or copy number gain was detected in half of the patients who achieved a response. Prexasertib also showed early signs of activity when added to olaparib in patients with HGSOC previously treated with a PARPi^[95]^.

PARPi were also evaluated along with WEE1 kinase inhibitors in preclinical models and in clinical trials. The WEE1 kinase prevents entry into mitosis by inhibiting CDK1 and CDK2. WEE1 inhibition causes CDK1 activation, resulting in cell cycle acceleration, early mitotic entry and mitotic catastrophe, particularly when combined with DNA damaging agents^[82]^. Simultaneous inhibition of PARP and WEE1 is highly toxic, but this can be mitigated by adopting a sequential treatment strategy. Normal cells have low replicative stress and DNA damage, so sequential therapy is effective for tumor cells but less toxic to normal cells^[96]^. In a recent clinical trial, the efficacy of adavosertib alone or in combination with olaparib was investigated in 80 patients with EOC and PARPi resistance. Patients treated with the combination have a greater CBR (89% *vs.* 63%) compared to WEEi alone, but the ORR was similar between the two arms (29% *vs.* 23%). Among exploratory analyses, *BRCA* mutations appeared to correlate with lower ORR (20% in the adavosertib-alone arm and 19% in the adavosertib/olaparib arm) compared to the *BRCAwt* subgroup (31% in the adavosertib-alone arm and a 39% in the adavosertib-olaparib arm). Data on the benefit according to the HRD status are not yet available. Translational analyses are ongoing to identify potential predictive biomarkers^[97]^.

A synergistic activity among PARPi and ATR inhibitors (ATRi) has been observed in different models of ovarian cancer^[98,99]^. G2-M checkpoint is lost with ATR inhibition, and as a result, cells with damaged DNA can proceed into the cell cycle, and run into mitotic catastrophe and apoptosis. In addition, PARP inhibition increases dependence on ATR/CHK1 to maintain genome stability^[98]^. A study in *BRCAwt*, CCNE1 amplified platinum-resistant ovarian cancer patient-derived xenograft (PDX) models showed that the combination of PARPi and ATRi results in tumor reduction and a significant increase in OS^[98]^. A phase 2 trial investigated the combination of olaparib and the ATRi ceralasertib to overcome PARPi resistance. In 13 patients with PARPi resistance, an ORR of 46% was observed with a PFS of 7.6 months^[99]^. Given these preliminary results, future clinical trials are also needed to investigate this combination in HRp EOC as a strategy to overcome intrinsic resistance to PARPi. Moreover, dose optimization trials are required to define the best schedule to overcome the important challenge of the hematological toxicity of the combinations of DDR and cell cycle checkpoint inhibitors.

Histone deacetylases (HDACs) and bromodomain and extraterminal (BET) proteins are involved in gene transcription. BET inhibition downregulates WEE and decreases the expression of TOPBP1, a protein required for the activation of ataxia-telangiectasia mutated (ATM) and ataxia telangiectasia and Rad3-related (ATR). Phosphatidylinositol-3 kinase (PI3K) inhibition decreases *BRCA1* expression, while MEK inhibition decreases RAD51foci. In addition, MEKi increases PARP1 expression and decreases angiogenesis. The inhibition of CDK12 and CDK1 decreases the expression of proteins involved in the homologous recombination. WEE and ATR inhibition results in the loss of the G2-M checkpoint, and cells with damaged DNA advance in mitotic phase. All these mechanisms lead to homologous recombination deficiency.

## TARGETING REPLICATION STRESS TO TREAT HRp TUMORS

CCNE and MYC amplified EOC have been identified as possible targets of RS response inhibitors, such as CHK1, ATRi, and WEE1 inhibitors (WEE1i). The inhibition of these checkpoints leads to inappropriate initiation of replication from multiple origins, depletion of replication factors, fork collapse, and cell death^[100]^. In CCNE1-amplified HGSOC cell lines, the CDK2 inhibitor dinaciclib suppresses cell growth and cell cycle progression, inducing apoptosis with a rise of intracellular radical oxygen species (ROS) levels^[101]^. The combination of a CDK2i and an AKTi also resulted in tumor regression in CCNE1-amplified cell lines of HGSOC^[102]^. In MYC overexpressing ovarian cancer xenograft models, the CDK4/6i, palbociclib induced tumor regression when combined with olaparib, showing a potential synergy among these two DDR targeting agents^[103]^.

Among the genes identified as related to a loss of fitness in CCNE amplified cells, there is PKMYT1, which encodes the protein kinase Myt1, involved in the negative regulation of CDK1. RP-6306, a highly selective inhibitor of this target, was tested on several ovarian carcinoma cell lines and showed greater toxicity in CCNE1 amplified preclinical models alone or with gemcitabine^[104]^. Following these promising preclinical results, RP-6306 enters clinical investigation as a single agent (NCT04855656) and in association with gemcitabine (NCT05147272).

The ATRi berzosertinib has been combined with gemcitabine in a phase II trial in patients with recurrent EOC and a platinum-free interval < 6 months (*n* = 70). PFS was improved in the experimental arm and the greatest benefit in PFS was obtained in patients with a platinum-free interval ≤ 3 months (PFS 27.7 weeks *vs.* 9 weeks, HR 0.29; 90%CI 0.12 to 0.71)^[105]^. A preplanned exploratory analysis revealed a better response trend with gemcitabine alone in patients in the Signature 3-negative subgroup (reflective of HRp tumors) compared to patients in the Signature3-positive subgroup (reflective of HRD tumor)^[106]^. In addition, tumors with high RS achieved a better response to gemcitabine, likely due to the gemcitabine-induced RS through inhibition of DNA repair and suppression of ribonucleotide reductase^[106]^.

Another promising agent in this setting is the WEE1i adavosertib, which has been investigated in different phase 2 trials in platinum-sensitive and resistance recurrent OC^[97,107-109]^. Interestingly, the preliminary results of a phase 2 study of single-agent adavosertib in CCNE1 overexpressed recurrent platinum-resistant or refractory HGSOC has been presented. The WEE1 inhibitor resulted in a high ORR (53%) and a clinical benefit rate of 61%, with some patients showing sustained responses over time. Translational analyses are ongoing to better define potential predictive biomarkers of WEE1i activity^[110]^.

## CONCLUSIONS

Targeting the HRR pathway through PARP inhibition has revolutionized the treatment of EOC. Clinical trials have proved that the HRp subgroup has a modest advantage with PARPi compared to the HRD population. Different genomic assays are now available to assess the presence of HRD. Although in most studies, HRD status detected by these assays has been shown to be able to discriminate the magnitude of PARPi benefit in *BRCA*wt EOC, these assays cannot reliably identify the group of HRp women with EOC that definitively derive no benefit from PARPi. HRD tests and mutational signatures, although promising, do not represent the dynamic changes in the functional status of HRD^[111]^.

Although a direct comparison within a randomized trial is currently lacking, the benefit of PARPi as first-line maintenance is comparable to that observed with bevacizumab in women with HRp EOC. Given that in the HRp population, no benefit was shown in adding olaparib to bevacizumab in first-line settings, bevacizumab could still represent a viable alternative for this subgroup of patients^[15]^. Pending further data from ongoing clinical trials comparing PARPi *vs.* antiangiogenic treatment in the HRp population, the decision on the best first-line strategy should be based on the toxicity profile of the two classes of drugs and patients’ comorbidities.

Different studies are ongoing to explore strategies to induce HRD and increase the sensitivity to PARPi, even in HRp tumors. Recently, new insights into the biology of HRp and platinum-resistant tumors are sustaining the development of new promising agents targeting the RS^[100]^. These new agents, alone or in combination, have shown preliminary signs of activity, but their use is challenged by the safety profile and the need to define optimal doses and schedules to maximize the clinical activity while minimizing the occurrence and severity of the adverse events.

## DECLARATIONS

### Copyright

© The Author(s) 2023.
